# Real‐World Effectiveness and Safety of Ravulizumab in Patients With Paroxysmal Nocturnal Hemoglobinuria: Evidence From the International PNH Registry

**DOI:** 10.1002/ajh.70268

**Published:** 2026-03-07

**Authors:** Alexander Röth, Christopher J. Patriquin, Jeff Szer, Louis Terriou, Ami S. Patel, Jesse Metzger, Philippe Gustovic, Jun‐ichi Nishimura, Robert A. Brodsky

**Affiliations:** ^1^ Department of Hematology and Stem Cell Transplantation, West German Cancer Center University Hospital Essen, University of Duisburg‐Essen Essen Germany; ^2^ Division of Medical Oncology and Hematology University Health Network Toronto Ontario Canada; ^3^ Department of Clinical Hematology Peter MacCallum Cancer Centre and the Royal Melbourne Hospital Melbourne Victoria Australia; ^4^ Service de Médecine Interne et d'Immunologie Clinique, CHU Lille Lille France; ^5^ Alexion, AstraZeneca Rare Disease Boston Massachusetts USA; ^6^ Parexel Newton Massachusetts USA; ^7^ Alexion, AstraZeneca Rare Disease Baar Switzerland; ^8^ Department of Hematology and Oncology Osaka University Graduate School of Medicine Osaka Japan; ^9^ Division of Hematology Johns Hopkins Medicine Baltimore Maryland USA

**Keywords:** C5, complement inhibition, paroxysmal nocturnal hemoglobinuria, ravulizumab, real‐world outcomes

## Abstract

Ravulizumab, a second‐generation complement component 5 inhibitor (C5i) derived from eculizumab, with improved pharmacokinetics, is the current standard‐of‐care treatment for patients with paroxysmal nocturnal hemoglobinuria (PNH), where available. Pivotal trials have demonstrated durable long‐term efficacy, safety, and improved survival, and increasing real‐world evidence is required. This analysis utilized data from the International PNH Registry (NCT01374360) to evaluate the real‐world effectiveness and safety of ravulizumab, and to assess baseline characteristics at treatment initiation. Adults (aged 18–65 years) enrolled in the registry as of July 1, 2024, who were treated with ravulizumab for ≥ 6 months were included and stratified by prior eculizumab use (eculizumab‐experienced and C5i‐naive). Ravulizumab treatment outcomes were assessed for up to 24 months and included change in lactate dehydrogenase (LDH) ratio from baseline, transfusion avoidance, and adverse event rates. Data for 203 eculizumab‐experienced and 23 C5i‐naive patients were analyzed. During treatment, LDH ratio was maintained near normal (< 1.5 × the upper limit of normal in both groups); 86.8% of eculizumab‐experienced and 76.5% of C5i‐naive patients were transfusion‐independent, and a low rate of major adverse vascular events was reported (eculizumab‐experienced, 0.4 per 100 person‐years; C5i‐naive, no events). These findings reinforce the pivotal trial outcomes and demonstrate the effectiveness and safety of ravulizumab in the real‐world setting, further supporting ravulizumab as the first‐line treatment of choice for patients with PNH, where available.

**Trial Registration:** International PNH Registry: NCT01374360

## 1 Introduction

Paroxysmal nocturnal hemoglobinuria (PNH) is a rare hematologic disorder characterized by uncontrolled terminal complement activation, leading to significant morbidity and mortality [1]. Symptoms and disease manifestations include abdominal pain, fatigue, hemoglobinuria, hemolytic anemia, organ damage, pulmonary hypertension, shortness of breath, and thrombotic events (TEs) [1]. The symptoms experienced can be debilitating and reduce the quality of life for patients with PNH [2].

Treating PNH with the terminal complement component 5 inhibitors (C5i) eculizumab (Soliris, Alexion Pharmaceuticals Inc., Boston, MA, USA) or ravulizumab (Ultomiris, Alexion Pharmaceuticals Inc., Boston, MA, USA), the current standard of care, has been shown to result in the control of terminal complement activity and intravascular hemolysis, a reduction in TEs and organ damage, and improved survival and quality of life for patients [3, 4]. Ravulizumab is a second‐generation inhibitor derived from eculizumab, with improved pharmacokinetics, and has been shown to achieve immediate, complete, and sustained terminal complement inhibition at an extended weight‐based dosing regimen (every 8 weeks vs. every 2 weeks for eculizumab). Clinical trials have demonstrated that ravulizumab has non‐inferiority and a comparable safety profile to eculizumab in patients with PNH [5, 6], as well as proven durable long‐term efficacy, safety, and improved survival for up to 6 years [7].

The International PNH Registry (NCT01374360) is the largest global real‐world database of patients with PNH; it provides data on the natural history and clinical course of PNH irrespective of treatment. The registry was designed to collect disease‐ and treatment‐related data that could inform optimal management of PNH. Previous analyses of the registry population have summarized baseline clinical characteristics and disease burden [8, 9] and provided long‐term real‐world data on the effectiveness of eculizumab for the treatment of patients with PNH for up to 14 years [10, 11]; however, real‐world data on ravulizumab remains limited.

The aims of this analysis were, first, to evaluate the real‐world effectiveness and safety of ravulizumab in eculizumab‐experienced and C5i‐naive patients with PNH enrolled in the International PNH Registry and, second, to characterize the demographic and clinical profile of patients at C5i treatment initiation.

## 2 Methods

### 2.1 Population

The International PNH Registry (NCT01374360) was a prospective, multicenter, global, observational study that included patients of any age with a clinical diagnosis of PNH and/or a detectable PNH clone. The institutional review boards for all participating centers approved the registry and all patients were required to provide written informed consent before study entry. The registry was overseen by an executive committee of international experts and sponsored by Alexion Pharmaceuticals Inc. Details of the registry design and patient population have previously been published [8, 9].

For the current analysis, the population included patients with PNH aged 18–65 years enrolled in the registry as of July 1, 2024, with a known date of birth, sex, enrollment date, and eculizumab/ravulizumab treatment status. Baseline was defined as the date of ravulizumab treatment initiation. Eculizumab‐experienced patients were defined as having been treated with eculizumab for ≥ 6 months, had switched to ravulizumab at or after registry enrollment, and had discontinued eculizumab within < 28 days of baseline. C5i‐naive patients were defined as having initiated ravulizumab on or after registry enrollment, had no prior experience with eculizumab or had discontinued eculizumab > 28 days prior to baseline, and had a lactate dehydrogenase (LDH) ratio of ≥ 1.5 × the upper limit of normal (ULN) at baseline. Patients were excluded if they switched treatment between ravulizumab and eculizumab more than once in the registry, had missing data on eculizumab or ravulizumab status, or had received treatment with ravulizumab for < 6 months.

### 2.2 Outcomes

Outcomes reported comprised: patient demographics; medical history and laboratory values at baseline; change from baseline in LDH ratio (defined as LDH concentration divided by ULN; used to express LDH levels as a ratio to the normal range), hemoglobin (Hb), and estimated glomerular filtration rate (eGFR) during ravulizumab treatment, assessed at 6‐, 12‐, 18‐, and 24‐month follow‐up; change in red blood cell (RBC) transfusion dependence and number of RBC units transfused from baseline to last recorded follow‐up; rate of major adverse vascular events (MAVEs; including TEs), meningococcal infections, and deaths prior to baseline and during ravulizumab treatment; and frequency and reasons for discontinuation of ravulizumab treatment.

### 2.3 Statistical Analysis

Descriptive statistical analyses were performed. Categorical variables were described using frequencies and percentages, and continuous variables were described using mean, standard deviation, median, and interquartile range. For each outcome of interest, analyses were conducted only on the patients who had available data. Crude and adjusted incidence rates were calculated via Poisson regression. Covariates were age at baseline, gender, and history of MAVEs at baseline.

## 3 Results

### 3.1 Patient Demographics and Baseline Characteristics

Clinical characteristics and real‐world ravulizumab effectiveness and safety data were available for 226 patients with PNH (eculizumab‐experienced, *n =* 203; C5i‐naive, *n =* 23). There were several notable differences in demographics and baseline clinical characteristics between the eculizumab‐experienced and C5i‐naive groups (Table 1). These included a higher proportion of females in the C5i‐naive group (69.6%) than in the eculizumab‐experienced group (50.2%), and a higher proportion of White/Caucasian patients in the eculizumab‐experienced group (68.8% vs. 47.8%). Compared with eculizumab‐experienced patients, a higher proportion of C5i‐naive patients had a history of bone marrow disorder (59.1% vs. 40.8%).

**TABLE 1:** Patient demographics and baseline^a^ characteristics.

	Eculizumab‐experienced (*n =* 203)	C5i‐naive^b^ (*n =* 23)
**Demographics**		
Geographic region, *n* (%)		
Europe	133 (65.5)	9 (39.1)
Asia	47 (23.2)	11 (47.8)
North America	18 (8.9)	3 (13.0)
Australia/New Zealand	5 (2.5)	0
Female, *n* (%)	102 (50.2)	16 (69.6)
Race, *n* (%)		
White/Caucasian	139 (68.8)	11 (47.8)
Asian	51 (25.2)	11 (47.8)
Black or African American	8 (4.0)	0
Other	4 (2.0)	1 (4.3)
Ethnicity, *n* (%)		
Not Hispanic/Latino	198 (98.0)	23 (100)
Hispanic/Latino	4 (2.0)	0
Age at ravulizumab initiation, years, median (IQR)	44.4 (36.3, 52.8)	48.6 (32.6, 60.1)
Time from disease start to ravulizumab initiation, years, median (IQR)	12.8 (7.7, 18.9)	5.7 (1.1, 12.0)
Duration of previous eculizumab treatment, years, median (IQR)	7.7 (4.4, 11.2)	NA
**Baseline medical history**		
History of bone marrow disorder, *n* (%)	82 (40.8)	13 (59.1)
Ongoing at baseline	51 (62.2)	11 (84.6)
Missing data	2	1
History of aplastic or hypoplastic anemia, *n* (%)	70 (35.2)	11 (50.0)
Missing data	4	1
History of MAVE, *n* (%)^c^	47 (23.3)	3 (13.0)
Missing data	1	0
History of TE, *n* (%)	38 (18.8)	2 (8.7)
Missing data	1	0
History of pulmonary hypertension, *n* (%)	8 (4.0)	2 (9.1)
Missing data	3	1
**Baseline laboratory values**		
LDH (U/L)		
Median (IQR)	257.0 (223.0, 330.0)	1234.0 (504.0, 1853.0)
Data available, *n*	154	23
LDH ratio (×ULN)		
Median (IQR)	1.0 (0.9, 1.2)	5.2 (2.3, 7.1)
Data available, *n*	147	23
LDH ratio, *n* (%)		
< 1.5 × ULN	131 (89.1)	0
≥ 1.5 × ULN	16 (10.9)	23 (100)
Data available, *n*	147	23
Hemoglobin (g/dL)		
Median (IQR)	11.1 (9.9, 12.5)	9.6 (8.8, 11.3)
Data available, *n*	145	22
eGFR (mL/min/1.73 m^2^)		
Median (IQR)	104.2 (88.2, 117.1)	97.6 (82.5, 119.2)
Data available, *n*	161	23
Percentage of GPI‐deficient granulocytes		
Median (IQR)	94.2 (80.0, 98.8)	85.0 (43.6, 97.8)
Data available, *n*	95	19

Abbreviations: C5i, complement component 5 inhibitor; eGFR, estimated glomerular filtration rate; GPI, glycosylphosphatidylinositol; IQR, interquartile range; LDH, lactate dehydrogenase; MAVE, major adverse vascular event; NA, not applicable; TE, thrombotic event; ULN, upper limit of normal.

^a^ Baseline was defined as the date of ravulizumab treatment initiation.

^b^ With an LDH ratio of ≥ 1.5 × ULN.

^c^ Includes both TE and non‐TE MAVEs.

The median duration of follow‐up was 2.5 and 1.3 years in eculizumab‐experienced and C5i‐naive patients, respectively (Table S1). Notably, the majority of eculizumab‐experienced patients (74.8%) had at least 24 months of follow‐up, with 44.8% followed for 24 to ≤ 36 months and 30.0% for more than 36 months. In contrast, follow‐up durations among C5i‐naive patients were more evenly distributed; 34.8% had 6 to < 12 months, 21.7% had 12 to < 18 months, 13.0% had 18 to < 24 months, 21.7% had 24 to ≤ 36 months, and only 8.7% had > 36 months of follow‐up.

### 3.2 Change in Laboratory Values up to the 24‐Month Follow‐Up

**FIGURE 1:**
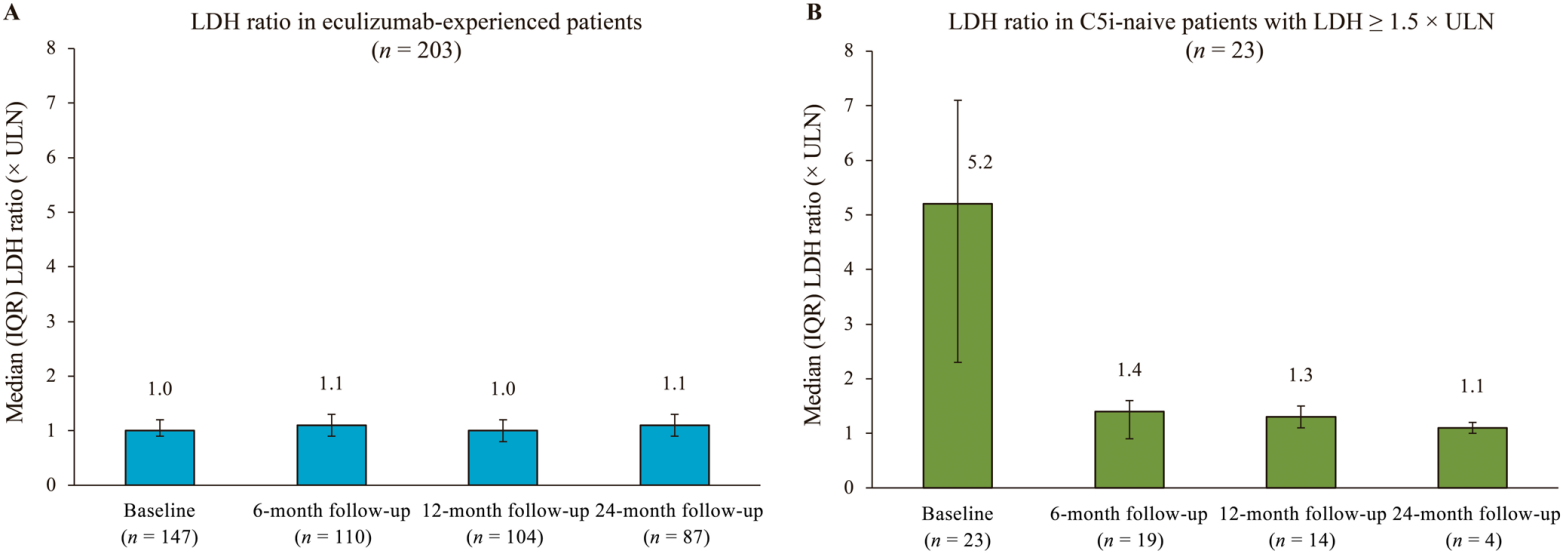
LDH ratio at specified time points in (A) eculizumab‐experienced patients and (B) C5i‐naive patients (with an LDH ratio of ≥ 1.5 × ULN). Baseline was defined as the date of ravulizumab treatment initiation. C5i, complement component 5 inhibitor; IQR, interquartile range; LDH, lactate dehydrogenase; ULN, upper limit of normal.

In eculizumab‐experienced patients, the LDH ratio was maintained between 1.0 and 1.1 × ULN from baseline to the 24‐month follow‐up (Figure 1). Laboratory values for Hb levels and eGFR also remained stable from baseline up to the 24‐month follow‐up in this group; Hb levels ranged from 11.1 to 11.2 g/dL (Table 2).

**TABLE 2:** Change in laboratory values following ravulizumab treatment initiation.

	Baseline^a^	6‐month follow‐up	12‐month follow‐up	24‐month follow‐up
**Eculizumab‐experienced** (***n** **=*** **203**)				
LDH (U/L), *n*	154	119	115	96
Median (IQR)	257.0 (223.0, 330.0)	268.0 (216.0, 348.0)	260.0 (209.0, 326.0)	257.5 (221.5, 338.5)
CfB,^a^ median (IQR)	NA	8.0 (−16.0, 38.0)	−3.0 (−29.0, 25.0)	10.0 (−30.0, 36.5)
LDH ratio (×ULN), *n*	147	110	104	87
Median (IQR)	1.0 (0.9, 1.2)	1.1 (0.9, 1.3)	1.0 (0.8, 1.2)	1.1 (0.9, 1.3)
CfB, median (IQR)	NA	0.0 (−0.1, 0.2)	0.0 (−0.1, 0.1)	0.1 (−0.1, 0.2)
Hemoglobin (g/dL), *n*	145	108	104	83
Median (IQR)	11.1 (9.9, 12.5)	11.2 (10.1, 12.5)	11.2 (9.9, 12.4)	11.2 (9.9, 12.6)
CfB, median (IQR)	NA	0.0 (−0.5, 0.6)	0.1 (−0.5, 0.7)	0.0 (−0.6, 0.6)
eGFR (mL/min/1.73 m^2^), *n*	161	121	120	101
Median (IQR)	104.2 (88.2, 117.1)	103.7 (89.5, 119.3)	102.9 (87.5, 118.8)	102.4 (88.3, 116.5)
CfB, median (IQR)	NA	−0.1 (−2.4, 4.9)	−0.8 (−4.6, 4.5)	−1.7 (−5.3, 4.4)
**C5i‐naive** ^b^ (***n** **=*** **23**)				
LDH (U/L), *n*	23	20	15	5
Median (IQR)	1234.0 (504.0, 1853.0)	275.5 (210.0, 340.5)	287.0 (209.0, 460.0)	232.0 (223.0, 268.0)
CfB, median (IQR)	NA	−917.0 (−1492.5, −278.5)	−1049.0 (−1768.0, −245.0)	−1049.0 (−1757.0, −939.0)
LDH ratio (× ULN), *n*	23	19	14	4
Median (IQR)	5.2 (2.3, 7.1)	1.4 (0.9, 1.6)	1.3 (1.1, 1.5)	1.1 (1.0, 1.2)
CfB, median (IQR)	NA	−3.5 (−5.3, −1.0)	−4.0 (−7.0, −1.0)	−5.3 (−6.8, −2.8)
Hemoglobin (g/dL), *n*	22	19	13	4
Median (IQR)	9.6 (8.8, 11.3)	11.3 (9.7, 12.1)	11.8 (11.0, 13.1)	11.0 (9.7, 12.2)
CfB, median (IQR)	NA	0.6 (−0.5, 1.9)	1.5 (−0.1, 2.4)	0.2 (−1.2, 1.1)
eGFR (mL/min/1.73 m^2^), *n*	23	19	12	3
Median (IQR)	97.6 (82.5, 119.2)	106.5 (72.3, 120.3)	110.1 (97.2, 118.2)	120.1 (102.2, 120.4)
CfB, median (IQR)	NA	−0.5 (−5.1, 3.0)	−3.6 (−7.1, 3.3)	−6.4 (−6.4, 3.7)

*Note: n* indicates the number of patients with a reported value.

Abbreviations: C5i, complement component 5 inhibitor; CfB, change from baseline; eGFR, estimated glomerular filtration rate; IQR, interquartile range; LDH, lactate dehydrogenase; NA, not applicable; ULN, upper limit of normal.

^a^ Baseline was defined as the date of ravulizumab treatment initiation.

^b^ With an LDH ratio of ≥ 1.5 × ULN.

Following ravulizumab initiation, C5i‐naive patients demonstrated a reduction in LDH ratio from baseline from 5.2 to 1.4 × ULN at the 6‐month follow‐up, which was further reduced to 1.1 × ULN at the 24‐month follow‐up (Figure 1). Laboratory values for Hb and eGFR increased from baseline to 6 months (Hb: from 9.6 to 11.3 g/dL; eGFR: from 97.6 mL/min/1.73 m^2^ to 106.5 mL/min/1.73 m^2^) and were maintained up to the 24‐month follow‐up (Table 2).

### 3.3 RBC Transfusions and Transfusion Avoidance

The proportion of patients in the eculizumab‐experienced group who had RBC transfusions decreased from the period 12 months prior to baseline (13.5%) to the 12 months following baseline (11.9%) (Table 3), and the rate of RBC transfusions did not change notably during this time period (1.0 [95% CI 0.9, 1.2] per‐person per‐year [PPPY] and 0.9 [95% CI 0.8, 1.0] PPPY, respectively).

**TABLE 3:** RBC transfusion status in eculizumab‐experienced and C5i‐naive patients.

	Eculizumab‐experienced (*n* = 203)	C5i‐naive^a^ (*n* = 23)
**RBC transfusions during 12 months prior to baselin** **e** ^b^		
Number of patients with reported value, *n*	200	23
Proportion of patients with RBC transfusions, *n* (%)	27 (13.5)	9 (39.1)
Total units transfused, *n*	202	71
Mean (SD)	1.0 (4.9)	3.1 (5.7)
Median (Q1, Q3)	0 (0, 0)	0 (0, 4.0)
Rate, units transfused PPPY (95% CI)	1.0 (0.9, 1.2)	3.1 (2.4, 3.9)
**RBC transfusions during 12 months post baselin** **e** ^b^		
Number of patients with reported value, *n*	201	23
Proportion of patients with RBC transfusions, *n* (%)	24 (11.9)	5 (21.7)
Total units transfused, *n*	179	19
Mean (SD)	0.9 (3.8)	0.8 (2.5)
Median (Q1, Q3)	0 (0, 0)	0 (0, 0)
Rate, units transfused PPPY (95% CI)	0.9 (0.8, 1.0)	0.8 (0.5, 1.3)

Abbreviations: C5i, complement component 5 inhibitor; CI, confidence interval; LDH, lactate dehydrogenase; PPPY, per‐person per‐year; Q1, first quartile; Q3, third quartile; RBC, red blood cell; SD, standard deviation; ULN, upper limit of normal.

^a^ With an LDH ratio of ≥ 1.5 × ULN.

^b^ Baseline was defined as the date of ravulizumab treatment initiation.

A decrease in the proportion of patients who received an RBC transfusion from 12 months prior to baseline (39.1%) to the 12 months following baseline (21.7%) was also observed in the C5i‐naive group, with a corresponding reduction in the rate of RBC transfusions (3.1 [95% CI 2.4, 3.9] PPPY and 0.8 [95% CI 0.5, 1.3] PPPY, respectively).

**FIGURE 2:**
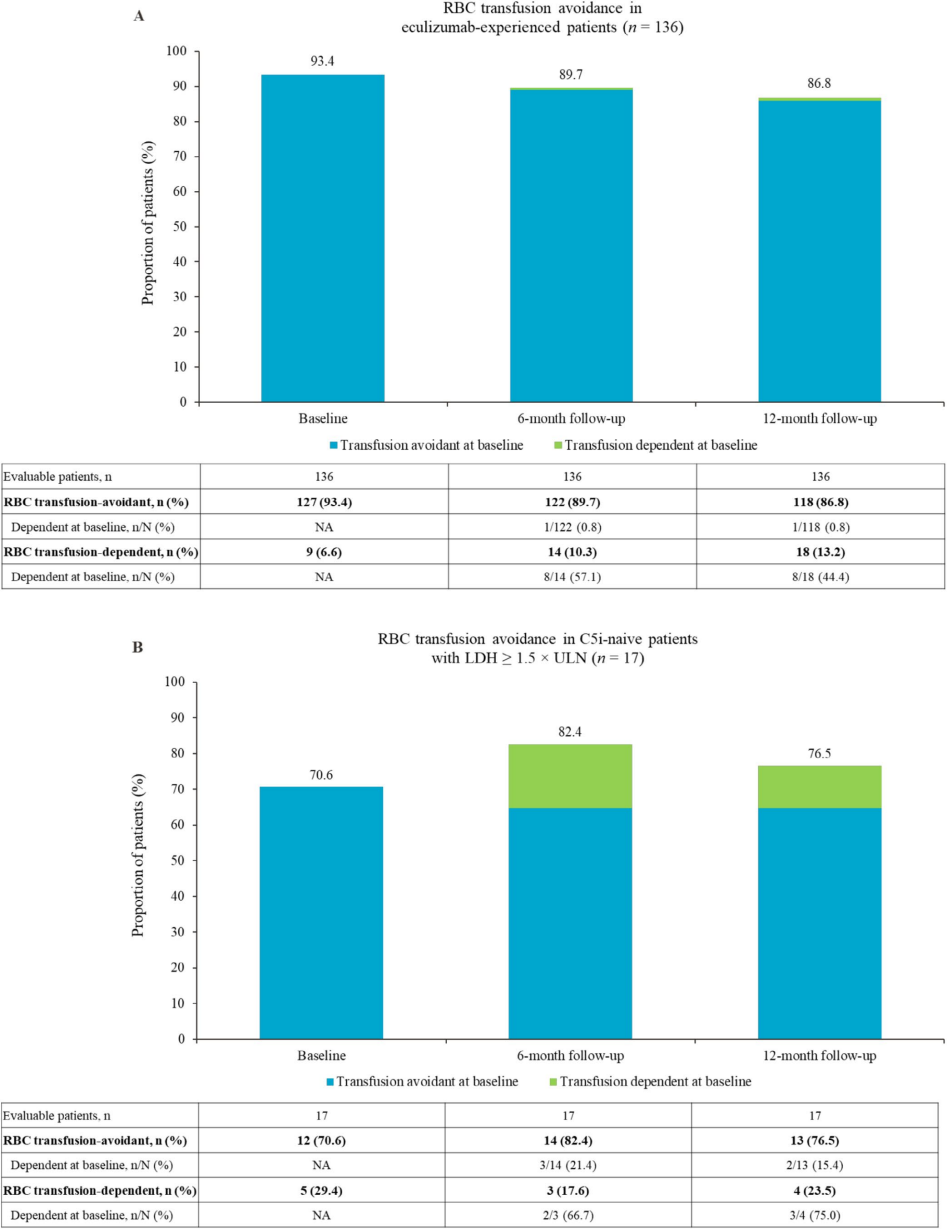
RBC transfusion avoidance at specified time points in (A) eculizumab‐experienced patients and (B) C5i‐naive patients (with an LDH ratio of ≥ 1.5 × ULN) with complete data. Baseline was defined as the date of ravulizumab treatment initiation. C5i, complement component 5 inhibitor; LDH, lactate dehydrogenase; NA, not applicable; RBC, red blood cell; ULN, upper limit of normal.

Overall, there was no notable change from baseline in the proportion of eculizumab‐experienced patients avoiding RBC transfusions at 6 and 12 months after ravulizumab initiation. The proportion of C5i‐naive patients avoiding RBC transfusions increased slightly from baseline at 6 months after ravulizumab initiation, which was maintained through to 12 months (Figure 2).

### 3.4 Ravulizumab Discontinuation

In total, 11 (5.4%) eculizumab‐experienced patients and 2 (8.7%) C5i‐naive patients discontinued treatment with ravulizumab (Table 4). In eculizumab‐experienced patients, the most common reasons reported for the discontinuation of ravulizumab were switching to another anticomplement treatment (*n =* 3) or switching back to eculizumab (*n =* 2); the reason for discontinuation was not reported in three patients. In C5i‐naive patients, reasons reported for the discontinuation of ravulizumab included switching to another anticomplement treatment (*n =* 1) and lack of efficacy (*n =* 1).

**TABLE 4:** Treatment discontinuations in eculizumab‐experienced and C5i‐naive patients.

Ravulizumab discontinuations, *n* (%)	Eculizumab‐experienced (*n =* 203)	C5i‐naive^a^ (*n =* 23)
Discontinued treatment	11 (5.4)	2 (8.7)
**Reported reason for discontinuation**		
Switch to IV eculizumab	2 (25.0)	0
Switch to other anti‐complement therapy^b^	3 (37.5)	1 (50.0)
Physician decision	1 (12.5)	0
Lack of efficacy	0	1 (50.0)
Adverse event	1 (12.5)	0
Death	1 (12.5)	0
Reason for discontinuation not reported	3 (37.5)	0

Abbreviations: C5i, complement component 5 inhibitor; IV, intravenous; LDH, lactate dehydrogenase; ULN, upper limit of normal.

^a^ With an LDH ratio of ≥ 1.5 × ULN.

^b^ Including patients who were enrolled in a clinical trial or were treated with bone marrow transplantation.

### 3.5 Safety

Overall, there was a reduction in both the number and rate of patients experiencing MAVEs and TEs following ravulizumab treatment initiation (Table 5). In the eculizumab‐experienced group, before receiving treatment with eculizumab (141.5 person‐years [PYs] of untreated time), nine patients (4.4%) had a history of MAVEs (nine events), of which five (2.5%) had experienced TEs (five events) (event rate: 6.4 and 3.5 per 100 PYs, respectively). During treatment with eculizumab (1583.1 PYs of exposure), MAVEs occurred in 13 patients (6.4%; 13 events), of which 11 had experienced TEs (5.4%; 11 events) (event rate: 0.8 and 0.7 per 100 PYs, respectively). After switching treatment to ravulizumab, one (0.5%) patient experienced a MAVE that was considered a TE (one event) (event rate: 0.4 per 100 PYs). In the C5i‐naive group, before receiving treatment with ravulizumab, two patients (8.7%) had a history of MAVEs (two events), of which one (4.3%) had experienced a TE (one event) (event rate: 2.7 and 1.4 per 100 PYs, respectively). No MAVEs or TEs were reported after commencing treatment with ravulizumab.

**TABLE 5:** Incidence of adverse events in eculizumab‐experienced and C5i‐naive patients.

	Eculizumab‐experienced (*n =* 203)			C5i‐naive^a^ (*n =* 23)	
	Untreated time^b^	Eculizumab treated time^c^	Ravulizumab treated time^d^	Untreated time	Ravulizumab treated time
Patients at risk^e^	91	202	202	19	23
**MAVEs** ^f^					
Patients with events (total events)	9 (9)	13 (13)	1 (2)	2 (2)	0 (0)
Person‐years	141.5	1583.1	552.5	73.5	35.8
Estimated rate^g^ per 100 person‐years (95% CI)	6.4 (3.3, 12.2)	0.8 (0.5, 1.4)	0.4 (0.1, 1.4)	2.7 (0.7, 10.9)	NA
**T** **Es** ^h^					
Patients with events (total events)	5 (5)	11 (11)	1 (2)	1 (1)	0 (0)
Details of TEs					
Pulmonary embolus	1	2	1	0	0
Thrombophlebitis/DVT	2	6	0	0	0
Hepatic/portal vein thrombosis	2	2	0	0	0
Cerebral venous occlusion	0	1	0	1	0
Person‐years	141.5	1583.1	552.5	73.5	35.8
Estimated rate per 100 person‐years (95% CI)	3.5 (1.5, 8.5)	0.7 (0.4, 1.3)	0.4 (0.1, 1.4)	1.4 (0.2, 9.7)	NA
**Meningococcal infections**					
Patients with events (total events)	0	3 (3)	0	0	0 (0)
Person‐years	141.5	1583.1	552.5	73.5	35.8
Estimated rate per 100 person‐years (95% CI)	NA	0.2 (0.1, 0.6)	NA	NA	NA
**Deaths**					
Patients with events (total events)	0 (0)	0 (0)	1 (1)	0 (0)	0 (0)
Person‐years	141.5	1583.1	552.5	73.5	35.8
Estimated rate per 100 person‐years (95% CI)	NA	NA	0.2 (0, 1.3)	NA	NA

Abbreviations: C5i, complement component 5 inhibitor; CI, confidence interval; DVT, deep vein thrombosis; MAVE, major adverse vascular event; NA, not applicable; TE, thrombotic event; ULN, upper limit of normal.

^a^ With an LDH ratio of ≥ 1.5 × ULN.

^b^ Prior to treatment with any C5i.

^c^ During eculizumab treatment, prior to ravulizumab initiation.

^d^ During ravulizumab treatment.

^e^ Patients at risk of MAVEs, TEs, or meningococcal infection.

^f^ Includes both TEs and non‐TE MAVEs. Non‐TE MAVEs include amputation (nontraumatic, nondiabetic), gangrene (nontraumatic, nondiabetic), myocardial infarction, transient ischemic attack, unstable angina, and other MAVEs.

^g^ Adjusted rates for MAVEs, TEs, meningococcal infections, and deaths are not presented owing to the sample size, multiple adjustment variables, and the very small number of events, particularly among the C5i‐naive population.

^h^ Includes acute peripheral vascular disease occlusion, cerebral arterial occlusion/cerebrovascular accident, cerebral venous occlusion, dermal thrombosis, hepatic/portal vein thrombosis, mesenteric/visceral arterial thrombosis, mesenteric/visceral vein thrombosis, pulmonary embolus, renal arterial thrombosis, renal vein thrombosis, and thrombophlebitis/deep vein thrombosis.

No meningococcal infections occurred in either group during ravulizumab treatment. One patient in the eculizumab‐experienced group died during ravulizumab treatment (event rate: 0.2 per 100 PYs); the cause of death was considered unrelated to ravulizumab treatment. The cause of death in this patient was renal cell carcinoma, which relapsed prior to ravulizumab initiation. There were no deaths in the C5i‐naive group.

## 4 Discussion

Despite limited real‐world evidence to date, this analysis of 226 patients from the International PNH Registry represents the largest international dataset on ravulizumab treatment in patients with PNH, which is helping to address an important evidence gap. The cohort included both C5i‐naive patients and those who had achieved disease control on eculizumab, representing a selected population of responders. These findings provide valuable insights into real‐world clinical outcomes, demonstrating sustained disease control with ravulizumab across both patient groups; results that are consistent with pivotal clinical trial data and recent UK‐real‐world experience [7, 12]. Importantly, no new safety signals were identified throughout the 24‐month follow‐up period.

Elevated LDH is a direct biochemical indicator of intravascular hemolysis, and a reliable surrogate marker for disease activity in PNH [8, 9]. Our analysis demonstrated that intravascular hemolysis was well controlled in both eculizumab‐experienced and C5i‐naive patients, with LDH ratios maintained between 1.0 and 1.4 × ULN and below the 1.5 × ULN threshold associated with an increased thrombotic risk [13]. Hb levels remained stable (11.0–11.8 g/dL) and renal function was preserved, with eGFR levels ≥ 90/mL/min/1.73 m^2^ throughout the 24‐month follow‐up [14].

In pivotal studies 301 and 302, similar proportions of eculizumab‐experienced and C5i‐naive patients with PNH treated with ravulizumab for 1 year achieved transfusion avoidance (84.8% and 72.0%, respectively) [15, 16], and a low rate of MAVEs (including TEs) were reported for up to 6 years (0.7 and 1.4 per 100 PYs, respectively) [7]. Our analysis aligned with these outcomes as transfusion avoidance was maintained or achieved in > 85% of eculizumab‐experienced patients and > 75% of C5i‐naive patients at 12 months of follow‐up. Similarly, the rate of MAVEs and TEs was low: 0.4 and 0.4 per 100 PYs for eculizumab‐experienced patients, respectively; no events reported among C5i‐naive patients.

Importantly, there were no meningococcal infections reported in eculizumab‐experienced or C5i‐naive patients during ravulizumab treatment. The latter may be attributable to mitigating strategies, such as meningococcal vaccination prior to ravulizumab therapy, in addition to reduced contact with individuals at high risk of infection because of greater infection awareness and COVID‐19 pandemic measures [17].

Across both groups, 5.8% of patients discontinued ravulizumab over the 2‐year treatment period. Reasons for discontinuation included switching back to eculizumab, switching to another anticomplement therapy, or lack of efficacy. One death was reported in the eculizumab‐experienced group; the cause of death was considered unrelated to treatment, and no deaths were reported in the C5i‐naive group.

The limitations of this study include those typically associated with registry analyses, such as their observational nature, incomplete data, and lack of active follow‐up. Furthermore, there was a small number of patients in the C5i‐naive group, resulting in limited data.

In conclusion, this study provides new evidence that, in a real‐world population, improvements in clinical outcomes with eculizumab are maintained with ravulizumab. This analysis provides the largest real‐world dataset to date on ravulizumab in PNH, reinforcing its effectiveness and safety across diverse patient populations and supporting its continued use in clinical practice as the first line treatment of choice, where available.

## Funding

This study was funded by Alexion, AstraZeneca Rare Disease.

## Ethics Statement

The protocol for the International PNH Registry was approved by an ethics committee for each site and was conducted in compliance with the International Council for Harmonisation Guideline for Good Clinical Practice.

## Consent

The International PNH Registry was approved by the institutional review boards, or equivalent, of all participating centers. Written informed consent was provided by all eligible patients prior to study entry.

## Conflicts of Interest

A.R.: *Alexion*, *AstraZeneca Rare Disease*, *Apellis Pharmaceuticals*, *BioCryst*, *Bioverativ*, *Novartis*, *Recordati*, *Sanofi*, *Sobi*: consultancy, honoraria. *Roche*: consultancy, honoraria, research funding. C.J.P.: *Alexion*, *AstraZeneca Rare Disease*, *Sobi*: consultancy, honoraria, other: clinical site investigator. *Amgen*, *BioCryst*, *Novartis*, *Roche*, *Takeda*: consultancy, honoraria. *Regeneron*: other: clinical site investigator. J.S.: *Alexion*, *AstraZeneca Rare Disease, Eli Lilly*, *Novartis*, *Sobi*: honoraria, membership of an entity's Board of Directors or advisory committees, speakers' bureau. L.T.: *Alexion*, *AstraZeneca Rare Disease*: consultancy, membership of an entity's Board of Directors or advisory committees. *Novartis*, *Sobi*: consultancy, honoraria. *Roche*: consultancy, honoraria, research funding. A.S.P. and P.G.: *Alexion*, *AstraZeneca Rare Disease*: employment. J.M.: *Parexel International*: employment. J.N.: *Alexion*, *AstraZeneca Rare Disease*: honoraria, membership on an entity's Board of Directors or advisory committees, research funding. *Chugai Pharmaceutical*, *Roche*: membership of an entity's Board of Directors or advisory committees. R.A.B.: *Alexion*, *AstraZeneca Rare Disease*: research funding.

## Supporting information


**Table S1:** Duration of follow‐up.

## Data Availability

Alexion, AstraZeneca Rare Disease will consider requests for disclosure of clinical study participant‐level data provided that participant privacy is assured through methods like data deidentification, pseudonymization, or anonymization (as required by applicable law), and if such disclosure was included in the relevant study informed consent form or similar documentation. Qualified academic investigators may request participant‐level clinical data and supporting documents (statistical analysis plan and protocol) pertaining to Alexion‐sponsored studies. Further details regarding data availability and instructions for requesting information are available in the Alexion Clinical Trials Disclosure and Transparency Policy at https://www.alexionclinicaltrialtransparency.com/data‐requests/.
