# Supplementary material for: Real‐World Effectiveness and Safety of Ravulizumab in Patients With Paroxysmal Nocturnal Hemoglobinuria: Evidence From the International PNH Registry
Source: Am J Hematol. 2026 Mar 7;101(5):939–48. doi: 10.1002/ajh.70268 (PMC13055122; doi:10.1002/ajh.70268)
Supplement: Supplementary file 1 — Table S1: Duration of follow‐up. [file AJH-101-939-s001.docx]

# Supplementary Materials

Table S1 | Duration of follow-up.

|  | **Eculizumab-experienced**  **(*n* = 203)** | **C5i-naive^a^**  **(*n* = 23)** |
| --- | --- | --- |
| Follow-up duration, years, median (IQR) | 2.5 (2.0, 3.9) | 1.3 (0.8, 2.4) |
| Follow-up duration by category, *n* (%) |  |  |
| 6 to < 12 months | 10 (4.9) | 8 (34.8) |
| 12 to < 18 months | 15 (7.4) | 5 (21.7) |
| 18 to < 24 months | 26 (12.8) | 3 (13.0) |
| 24 to ≤ 36 months | 91 (44.8) | 5 (21.7) |
| > 36 months | 61 (30.0) | 2 (8.7) |

^a^With an LDH ratio of ≥ 1.5 × ULN.
Abbreviations: C5i, complement component 5 inhibitor; IQR, interquartile range; LDH, lactate dehydrogenase; ULN, upper limit of normal.
